# Bilateral congenital absence of patella

**DOI:** 10.4103/0019-5413.40264

**Published:** 2008

**Authors:** J Terrence Jose Jerome, Mathew Varghese, Balu Sankaran, Simon Thomas

**Affiliations:** Department of Orthopaedics, St Stephen's Hospital, New Delhi, India

**Keywords:** Absence, bilateral, congenital, patella, rare

## Abstract

Absence of patella as an isolated anomaly is extremely rare. It is usually absent as part of a syndrome, most commonly hereditary arthro-onchyo-dysplasia (Nail Patella Syndrome). We present a case of congenital bilateral absence of patella in a nine-year-old boy with no associated anomalies.

## CASE REPORT

A nine-year-old boy presented to our outpatient department with complaints of deformity in both knees, inability to straighten the knee, difficulty in standing and walking. On further questioning, the parents gave history of full term born, normal delivered child, with deformed knees since birth. Partially flexed knees noted at the time of birth, gradually worsened from the age of one and a half years, when he started walking. Deformity was more on the left knee, which made it difficult for him to walk.

**Figure 1:**
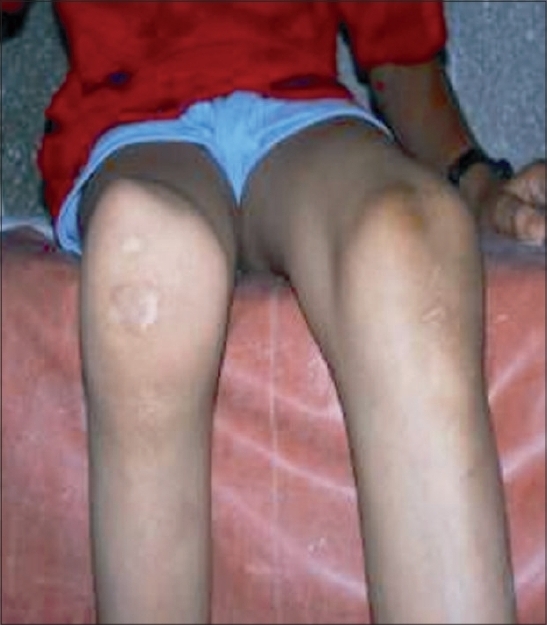
Clinical picture of the patient shows prominent femoral condyles with a hollow sulcus in between the condyles and bilateral absence of patella

**Figures 2 and 3:**
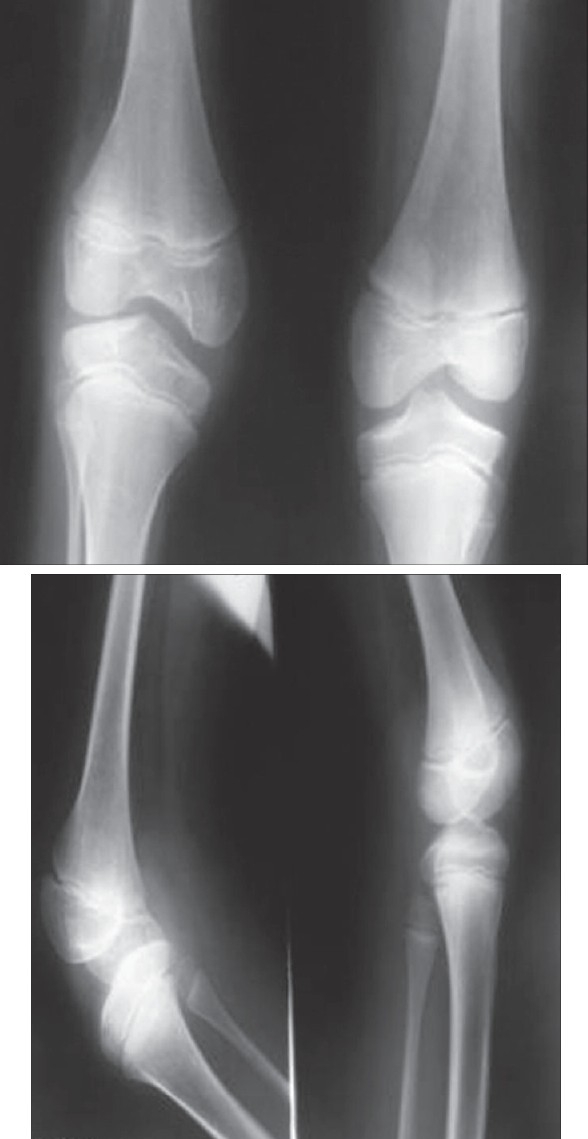
Radiograph shows the absence of both patellae and large femoral condyles in antero-posterior and lateral views

On examination there was bilateral absence of patella. Femoral condyles were prominent. Absence of both patellae and a hollow sulcus seen in between femoral condyles were the hallmark clinical features [Figure 1]. Femoral condyles were large and prominent. Fixed flexion deformity in knee measuring 80 degrees on the left side and 30 degrees on the right side was noted. The power of quadriceps hamstrings and gluteus maximus were good. Nails were clinically normal. No other deformity was present in the patient. Examination of upper limbs and other systems was clinically normal. His blood parameters were within normal limits. Ultrasound abdomen was normal. No renal abnormalities were noted. Radiographs of both knees [Figures 2 and 3] showed absence of both patellae and flexion deformity more on the left side.

**Figure 4:**
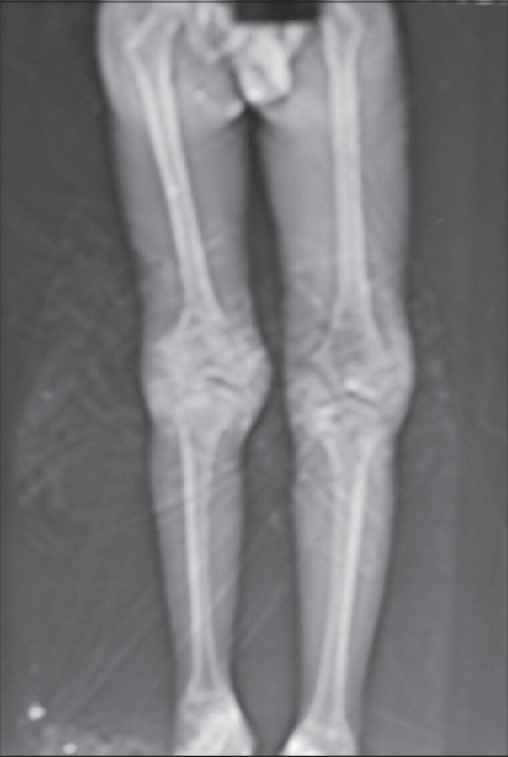
CT scanogram done to determine the limb length and other associated anomalies. There was no limb length discrepancy

Computerized axial tomography [Figure 4] was done to measure the limb length discrepancy and to look for other associated disorders in both lower limbs. Limb lengths were equal on both sides. No femur or tibial anomalies were seen.

**Figure 5:**
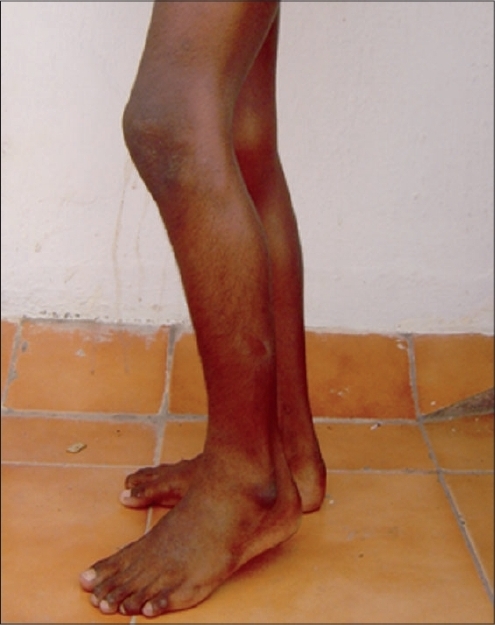
Postoperative picture shows a minimal residual flexion deformity in the left knee and compensatory ankle equinus

Left side fractional hamstring lengthening was done under spinal anesthesia. Sutures were removed after two weeks. Active and passive resisted physiotherapy was started after six weeks. Gait training was given. The patient was on regular follow-up every two months a year. Patient was 13 years old at the time of last follow-up. He can now walk with a bipedal independent gait with minimal flexion at the left knee and mild equinus at ankle [Figure 5]. He can also do his day-to-day activities, play, squat, sit cross legged and can lead a near normal life.

## DISCUSSION

Isolated absence of patella is extremely rare.^1^–^5^ It usually causes no disability to the patient. Congenital absence of patella is only one of several anomalies such as nail patella syndrome,^2^,^3^ dislocation of the knee, genu recurvatum, anomalies of the femur and fibula, clubfoot or dislocation of the hip, dystrophy in thumb nails, bifid thumb nails, decrease in the length of nails, hypoplastic patella,^7^ recurrent lateral dislocation of patella, genu valgum, slip of medial tibial plateau, cubitus valgus, hypoplasia of elbow with decreased range of motion, iliac horns,^8^ flaring of iliac crests with prominence of anterior superior iliac spines, and pelvic abnormalities. In these instances the treatment of any disability about the knee depends primarily on its chief cause, for example, genu recurvatum.^5^ It is this anomaly that should be treated; the absence of patella is of minor importance and requires no specific treatment.^5^ In a series reported by Guidera and associates, approximately 50% of children with nail patella syndrome underwent knee surgery to treat instability.^9^

Congenital absence of patella without any other osseous anomaly is accompanied by severe lateral dislocation of the extensor mechanism.^5^–^7^,^9^,^10^ In these instances placing the mechanism in the groove between the femoral condyles, transplanting the tibial tuberosity medially and transferring one or more of the medial hamstrings tendons to the extensor mechanism usually results in satisfactory function. The femoral condyles and the tibial tuberosity are often larger than normal. The quadriceps is usually strong and the extensor mechanism is well developed and glides in the patellar groove between the femoral condyles.^2^,^5^

Our patient had severe flexion deformity in his left knee, which made it difficult for him to walk. Often at times, he required support to walk and climb stairs. Conventional hamstring lengthening helped this patient in correcting the flexion deformity.^5^ Pre-operatively the flexion deformity was almost corrected with few degrees of residual flexion [Figure 5]. Patient was put on regular physiotherapy, which gradually straightened the knee without compromising the other muscle functions. No surgery was done for right knee. Deformity became supple after regular stretching exercises. Patient now walks with bipedal independent gait and a minimal flexion deformity in his left knee.

## CONCLUSIONS

- Isolated congenital bilateral absence of patella-extremely rare
- Not reported in the literature
- No major disability to the patient
- Treatment directed towards associated anomalies
